# Defining the Role of Medication Adherence in Poor Glycemic Control among a General Adult Population with Diabetes

**DOI:** 10.1371/journal.pone.0108145

**Published:** 2014-09-26

**Authors:** Becca S. Feldman, Chandra J. Cohen-Stavi, Morton Leibowitz, Moshe B. Hoshen, Shepherd R. Singer, Haim Bitterman, Nicky Lieberman, Ran D. Balicer

**Affiliations:** 1 Clalit Research Institute, Chief Physician's Office, Clalit Health Services, Tel Aviv, Israel; 2 Department of Medicine, New York University School of Medicine, New York, New York, United States of America; 3 Division of Epidemiology, Israeli Ministry of Health, Jerusalem, Israel; 4 The Ruth and Bruce Rappaport Faculty of Medicine, Technion, Israel Institute of Technology, Haifa, Israel; 5 Community Medicine Division, Clalit Health Services, Tel Aviv, Israel; 6 Department of Public Health, Faculty of Health Sciences, Ben-Gurion University of the Negev, Beer-Sheva, Israel; Emory University, United States of America

## Abstract

**Aims:**

This study assesses the attributable impact of adherence to oral glucose medications as a risk factor for poor glycemic control in population subgroups of a large general population, using an objective medication adherence measure.

**Methods:**

Using electronic health records data, adherence to diabetes medications over a two-year period was calculated by prescription-based Medication Possession Ratios for adults with diabetes diagnosed before January 1, 2010. Glycemic control was determined by the HbA1c test closest to the last drug prescription during 2010–2012. Poor control was defined as HbA1c>75 mmol/mol (9.0%). Medication adherence was categorized as “good” (>80%), “moderate” (50–80%), or “poor” (<50%). Logistic regression models assessed the role medication adherence plays in the association between disease duration, age, and poor glycemic control. We calculated the change in the attributable fraction of glucose control if the non-adherent diabetic medication population would become adherent by age-groups.

**Results:**

Among 228,846 diabetes patients treated by oral antiglycemic medication, 46.4% had good, 28.8% had moderate, and 24.8% had poor adherence. Good adherence rates increased with increasing disease duration, while glycemic control became worse. There was a strong inverse association between adherence level and poor control (OR = 2.50; CI = 2.43–2.58), and adherence was a significant mediator between age and poor control.

**Conclusions:**

A large portion of the diabetes population is reported to have poor adherence to oral diabetes medications, which is strongly associated with poor glycemic control in all disease durations. While poor adherence does not mediate the poorer glycemic control seen in patients with longer-standing disease, it is a significant mediator of poor glycemic control among younger diabetes patients. A greater fraction of poorly controlled younger patients, compared to older patients, could be prevented if at least 80% adherence to their medications was achieved. Therefore, our results suggest that interventions to improve adherence should focus on this younger sub-group.

## Introduction

Diabetes and its complications remain a major concern in health care management and clinical practice, with many patients unable to achieve target glycemic levels [1]. Poor disease control has been found to be associated with microvascular complications [2]–[4] and mortality [5]–[6]. Current recommendations for glycemic control targets, as assessed by HbA1c levels, are dependent on age and duration of illness [7]. Nevertheless, HbA1c levels greater than 75mmol/mol (9.0%) are universally considered poor control [8]. Despite evidence of the efficacy of hypoglycemic medications to help diabetes patients regulate and control their glucose levels, great variation in adherence to these medications has been reported, with studies often indicating poor or low average levels of adherence [9]–[15]. Consequently, the clinical focus of therapeutic interventions for improving glycemic control is often on oral medication adherence, and, at later disease stages, on adherence with recommended parenteral insulin treatment.

A substantial body of literature has demonstrated a positive association between medication adherence and glucose control [11], [16]–[22]; however, the methodologies of these studies have weaknesses that include small sample sizes, selective populations, and subjective patient reported measures–which potentially limit statistical significance and generalizability of results [23]–[24]. Previous studies have found that the young diabetes population [5], [17], [25]–[26] and individuals with long-term diabetes are at an increased risk for poor control [25], [27]–[28]; however, the factors contributing to this relationship are not well understood. Of those diabetes patients with longer duration of illness, many are taking multiple diabetes medications that are being actively managed by the patients or by their physicians. Multiple drug regimens pose a challenge to measuring medication adherence because of the potential for missing changes in the prescribed treatment regimen.

While the younger adult and long disease duration sub-groups have been identified as key population segments at-risk for poor glycemic control, the evidence of the extent to which medication adherence contributes to this poor control is still lacking. The present study aimed to retrospectively assess the importance of adherence to multiple diabetes medications in a large, general population of individuals with diabetes using an objective medication adherence measure that accounts for these multi-drug regimens. We evaluated this by testing whether medication adherence is an intermediary factor leading to poor glycemic control in different sub-segments of the general diabetes population.

## Methods

### Setting

Health care in Israel is universal and delivered primarily through four nationwide health plans, which act both as providers and insurers. All citizens are guaranteed a legally mandated minimum package of medical services [29]. Clalit Health Services (CHS), the largest of the health plans, insures approximately 4 million people (53% of the Israeli population) and provides hospital and community-based medical services including diagnostic procedures, medical treatments, laboratory testing, and hospitalizations. Members of CHS have a strong incentive to access primary care and obtain prescription medications within the CHS system because primary care visits are free of charge to members and medications are included in the benefit package with only nominal co-payments required. Integrated clinical electronic health records and administrative information on all CHS members are compiled in a comprehensive database linking data from hospitals, community-based clinics, laboratory and diagnostic testing offices, and CHS pharmacies. This study was approved by the Meir Hospital Institutional Review Board Ethics Committee for Clalit Organizational Studies. De-identifiable data were analyzed anonymously.

### Study population

**Figure 1:**
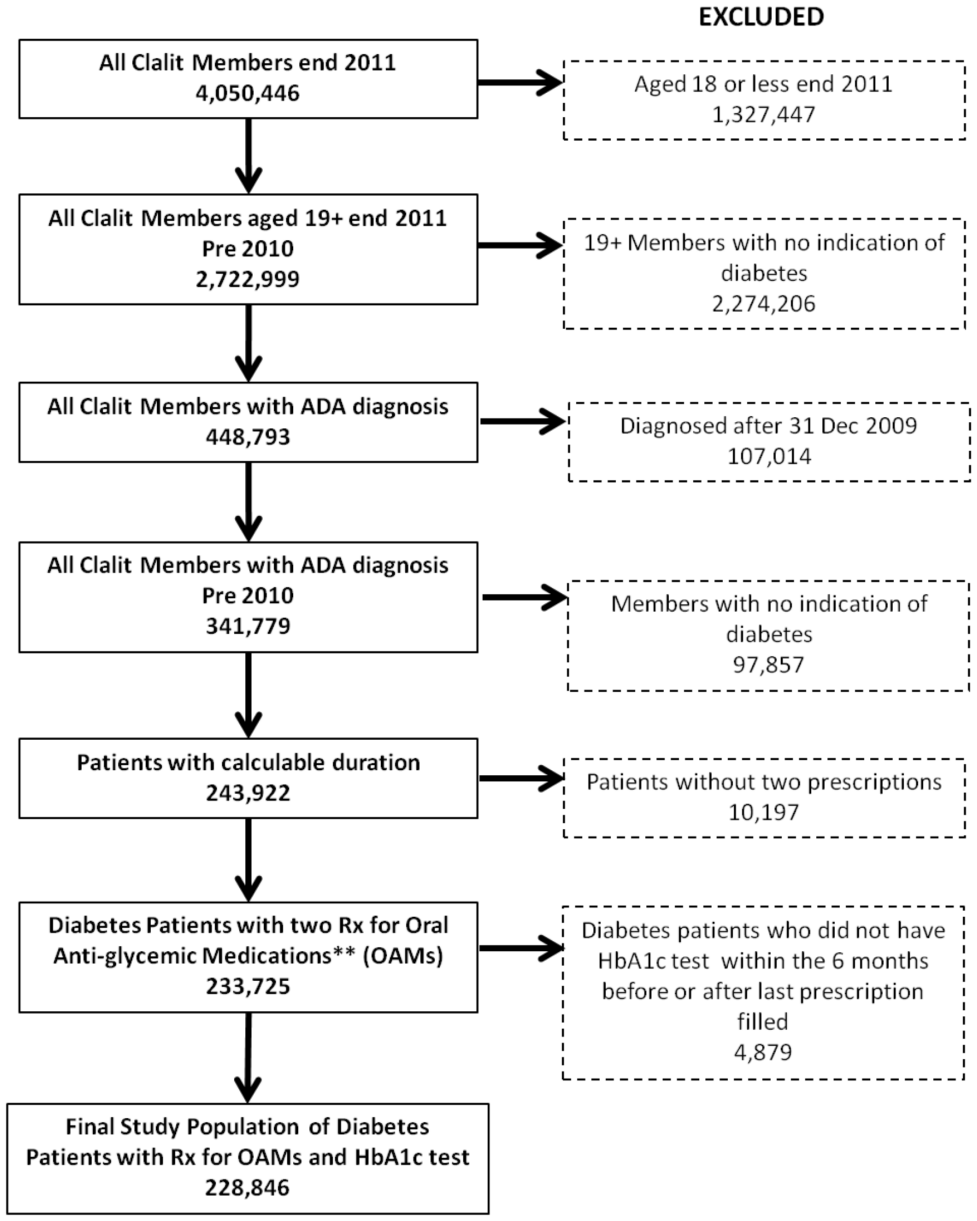
Patient exclusions flow chart. The figure shows the process for arriving at the final sample size. After all exclusion criteria were applied, a final study population of 228,846 patients with diabetes who had a prescription for oral anti-glycemic medications and an HbA1c test performed was yielded.

This study included all CHS members 19 years and older who were continuous members of CHS for the duration of the study period and were diagnosed with diabetes, based on criteria defined by the American Diabetes Association, on or before December 31, 2010. Those included had to have received written prescriptions for any of five types of oral antiglycemic medication (OAM) classes (biguanides, sulphonamides, dipeptidyl peptidase 4 inhibitors [DPP4], repaglinide or exenatide/liraglutide) a minimum of two times, at least 30 days apart, between January 1, 2010 and December 31, 2011 (Figure 1). The first medication prescription was defined as the index date. Members were included if they had an HbA1c test taken no more than six months preceding or following the last prescription they received during the two-year study period. We excluded members who had a hospitalization of 30 days or more during the study period, as we have no prescription information during this time. If a member died during the study period, the date of death was considered the end of the study, with adherence calculated for this period and the HbA1c level closest to the mortality date within the preceding six months recorded.

### Outcome

The primary outcome, study end-HbA1c, was defined as the HbA1c closest to the last prescription date in the study period, with a ceiling of 180 days before or after that date. Among new medication users, HbA1c measurements were included only if they were taken at least 30 days after the medication index date. Poor glucose control was defined as HbA1c>75 mmol/mol [9.0%].

### Covariates

#### Demographic and clinical data

Age (19–30, 30–54, 55–64, 65–74, and 75+), gender, population sector, and SES were collected at the index date. Age groups were created in order to achieve a fairly even distribution of the study population, except for the 19–30 age group. Population sector (Jewish and ethnic minority) and socio-economic status (SES; high, medium, and low) are defined at the clinic level. The BMI calculation was based on height and weight measurements (kg/m^2^) within one year before or after the index date. BMI was grouped into five categories: <22, 22–25, 25.1–30, 30.1–35 and>35. Insulin users were those patients who procured at least three insulin prescriptions in the six months leading up to the medication end date. Smoking was not included in the models due to high rates of missing values.

#### Primary predictor

Adherence to oral hypoglycemic medications was evaluated between January 1, 2010 and December 31, 2011 using the CHS repository of dispensing and prescription data (all prescriptions in CHS are electronic). Adherence was measured from the date of the first written OAM prescription (index date) until the day before the last such written prescription (end date). Adherence to individual OAM classes was measured using a medication possession ratio that integrates prescribed with dispensed medication data (MPRp) [30]. Specifically, the number of days that medication was *dispensed* is divided by the number of days between when the first and the last medication was *prescribed* [30]. As almost half of the diabetes patients in the sample were on more than one OAM, we aggregated adherence to medications into a single, representative measure, by deriving a “mean weighted adherence” (MWA) measure. The MWA for each member is the average of all of the individual MPRp's, weighted by the duration over which each was prescribed during the study period. MWA was categorized as ‘poor adherence’ (<50% adherent), ‘moderate adherence’ (50–79.9% adherent), and ‘good adherence’ (adherent 80%+). Initially, adherence was categorized into four groups, 0–20%, 20–50%, 50–80% and 80%+; however, because of the skewness of the MWA distribution towards greater adherence (>50%), the two lower groups were merged to create three adherence groups of more equal size. Good adherence was defined as≥80%, as seen in MPR-based adherence studies [6]. Duration of disease was measured from the date criteria for diabetes were first met until the study index date, and was categorized as ‘short’ (0–35 months), ‘intermediate’ (36–59 months) and ‘long’ (60+ months). Diabetes diagnosis data in our database was available from January 1, 2002 onwards, therefore limiting disease duration in our analysis to ten years. The grouping ‘long’ duration of illness is limited due to inconsistencies in diabetes incidence data records in the database prior to 2007. Before 2007, diabetes diagnoses were entered into the system in clusters over several years, or were entered as an ongoing disease, not a new diagnosis.

### Statistical analysis

We assessed individuals' medication adherence during a two-year window using MWA. The population was classified into two groups of HbA1c control (HbA1c ≤75 mmol/mol [9.0%] and>75 mmol/mol). Descriptive statistics of the independent variables were calculated for the overall population of members with diabetes and stratified by the two HbA1c control groups. Univariate logistic regression models were then generated to assess the unadjusted association of each of the independent variables with poor glucose control, as measured by HbA1c. All variables tested were significant and were included in the multivariate model.

Four multivariate logistic regression models were examined. The first was an adjusted full model to assess and further confirm the association between the two primary predictors (disease duration and medication adherence groups) and glucose control. In the second and third models, we sought to further understand the role of adherence to medication in the association between disease duration and glucose control. In the second model we checked whether adherence to medication was a mediator between disease duration and glucose control, and in the third model we included an interaction term of disease duration and adherence to medication to determine if the association between these factors differed among disease duration subgroups. Due to the significant interaction term, we stratified the full model to further examine the odds ratios of adherence to medications and glucose control by the three disease duration groups. We tested a fourth model to assess if adherence to medications mediates the association between age and glucose control. A test for trend was conducted using one-way ANOVA.

Lastly, we determined the population attributable fraction of glucose control if the poor-adherence population would become adherent to their diabetic medications across the five age groups. This adjusted analysis was performed by the standard method:

*Population attributable risk*  =  (*exposure* * [OR – 1])/(1 + *exposure* * [OR – 1])

## Results

The study population consisted of 228,846 (Figure 1) members with OAM-treated diabetes aged 18 and over with an average age of 65 years (SD 12). Among them, 19.3% (44,185) were also taking insulin (Table 1). Overall, 46.4% of the population had good adherence (MWA>80%), 28.8% had moderate adherence (MWA of 50-80%), and 24.8% had poor adherence (MWA<50%). The majority of the population (70.1%) had diabetes for>5 years (defined as long duration), and 14.9% had diabetes for <3 years (short duration).

**Table 1:** Demographic and clinical characteristics according to glucose control.

Descriptive Statistics of the Total Study Population									
		Totals		Glucose control - HgbA1c levels					
				≤75 mmol/mol (9.0%)			>75 mmol/mol (9.0%)		
		Total (N)	Total (%)	Mean	N	Percent %	Mean	N	Percent %
**Total**		228,846	100%		190,619	83.3%		38,227	16.7%
**Gender**	Women	117,805	51.5%		99,019	51.9%		18,786	49.1%
	Men	111,041	48.5%		91,600	48.1%		19,441	50.9%
**Age (avg)**		65		66			59		
**Age Categories**	<30	673	0.3%		424	0.2%		249	0.7%
	30–54	44,483	19.4%		31,373	16.5%		13,110	34.3%
	55–64	67,150	29.3%		54,608	28.6%		12,542	32.8%
	65–74	61,860	27.0%		54,111	28.4%		7,749	20.3%
	75+	54,680	23.9%		50,103	26.3%		4,577	12.0%
**BMI (avg)**		29.6		29.5			30.1		
**BMI Categories (kg/m^2^)**	<22	11,042	4.8%		9,201	4.8%		1,841	4.8%
	22–25	33,695	14.7%		28,677	15.0%		5,018	13.1%
	25.1–30	87,099	38.1%		73,570	38.6%		13,529	35.4%
	30.1–35	59,587	26.0%		49,118	25.8%		10,469	27.4%
	>35	33,738	14.7%		27,073	14.2%		6,665	17.4%
**Ethnicity**	Jew	182,652	79.8%		157,796	82.8%		24,856	65.1
	Ethnic minority	46,194	20.2%		32,823	17.2%		13,371	35.0%
**Socio-economic Status**	Not Low SES	128,422	56.1%		111,615	58.6%		16,807	44.0%
	Low SES	100,424	43.9%		79,004	41.4%		21,420	56.0%
**Insulin use**	Not using insulin	184,661	80.7%		163,873	86.0%		20,788	54.4%
	Insulin user	44,185	19.3%		26,746	14.0%		17,439	45.6%
**Diabetes Disease Duration**	1–35 mo*	34,210	14.9%		30,821	16.2%		3,389	8.9%
	36–59 mo*	34,249	15.0%		29,927	15.7%		4,322	11.3%
	60 mo+*	160,387	70.1%		129,871	68.1%		30,516	79.8%
**Weighted Medication Adherence**	Poor	56,817	24.8%		41,680	21.9%		15,137	39.6%
	Moderate	65,947	28.8%		53,876	28.3%		12,071	31.6%
	Good	106,082	46.4%		95,063	49.9%		11,019	28.8%

*mo  =  months.

In the descriptive analysis, patients with poor glucose control (HbA1c>75 mmol/mol [9.0%]) comprised 16.7% (N = 38,227) of the study population. This group was more likely to be younger (mean age was 59 years) compared to those who did not have poor glucose control (mean age was 66 years). There were also a higher proportion of members of low SES, ethnic minorities, insulin users, and those with a long duration of diabetes in the poorly controlled group (Table 1). The percentage of those with poor glucose control increased over disease duration, and nearly 80% of those with poor control had long duration diabetes (>5 years) as opposed to 70% in the entire study population. Despite this trend, a clear inverse relationship is seen between age and poor control, with the proportion of poorly controlled diabetes patients progressively decreasing with each increasing age group. Diabetes patients with poor glucose control were more likely to have poor medication adherence (39.6% vs. 21.9%) than those who did not have poor control (Table 1).

In the adjusted model, the following demographic characteristics were positively associated with poor glucose control: male gender (OR = 1.13, CI: 1.10–1.15), low SES (OR = 1.31; CI: 1.27–1.34), and being from the ethnic minority sector (OR = 1.46; CI: 1.42–1.51) (Table 2). Longer and intermediate disease duration were positively associated with poor control with OR = 2.48 (CI: 2.38–2.59) and OR = 1.46 (CI: 1.39–1.54), respectively, compared to those with short disease duration.

**Table 2:** Multivariable Regression Analysis for Poor Glycemic Control (HbA1c>75 mmol/mol [9.0%]).

Multivariable Analysis for Poor Glycemic Control (HbA1c>75 mmol/mol [9.0%])				
	B	OR	95% CI	
			Lower	Upper
Age 75 + (reference)				
Age <30	1.41	4.09	3.42	4.88
Age 30–54	1.28	3.59	3.45	3.74
Age 55–64	0.76	2.15	2.06	2.23
Age 65–74	0.30	1.35	1.29	1.40
Male	0.12	1.13	1.10	1.15
BMI>35 kg/m^2^ (reference)				
BMI <22 kg/m^2^	0.11	1.11	1.04	1.18
BMI 22–25 kg/m^2^	−.07	.93	.89	.97
BMI 25.1–30 kg/m^2^	−.11	.90	.86	.93
BMI 30.1–35 kg/m^2^	−.05	.95	.91	.99
Ethnic minority	0.38	1.46	1.42	1.51
Low SES*	0.27	1.31	1.27	1.34
Weighted Medication Adherence – Good (reference)				
Weighted Medication Adherence – Poor	0.92	2.50	2.43	2.58
Weighted Medication Adherence –Moderate	0.50	1.65	1.61	1.70
Insulin User	1.41	4.08	3.98	4.19
Diabetes Disease Duration – Short (1–35 mo**)				
Diabetes Disease Duration – Intermediate (36–59 mo**)	.38	1.46	1.39	1.54
Diabetes Disease Duration – Long (60 mo+**)	.91	2.48	2.38	2.59

*SES  =  socio-economic status.

**mo  =  months.

Adjusting for all covariates, poor medication adherence (OR = 2.50; CI: 2.42–2.58), as well as moderate adherence (OR = 1.65; CI: 1.61–1.70), were positively associated with poor control as compared to those with good adherence.

**Figure 2:**
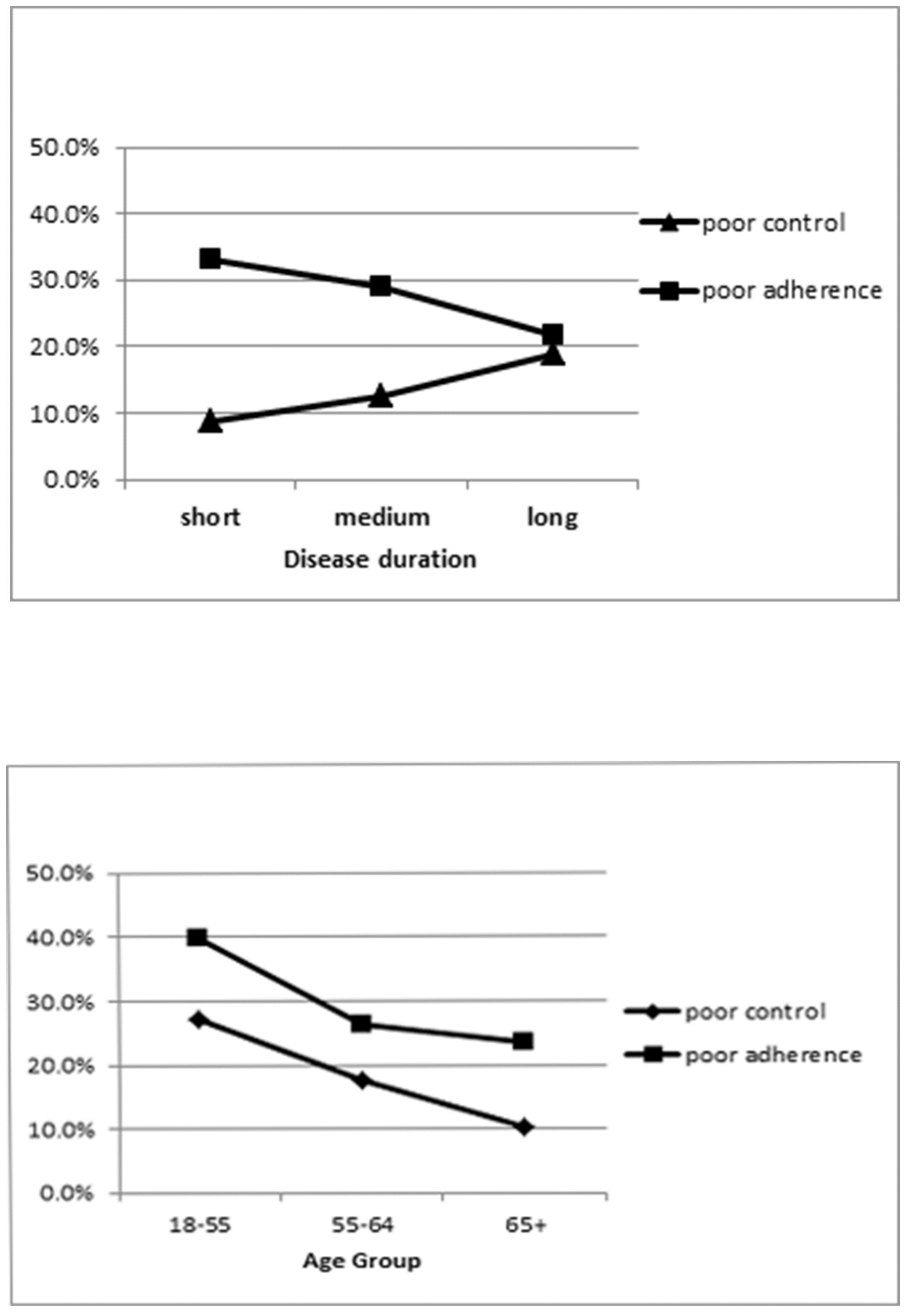
Percent of study population with poor adherence and poor control by disease duration and age. Figure 2a shows that there is a positive correlation between the duration of having diabetes, and the level of poor control over the disease. In other words, the longer a patient has diabetes, the poorer his control may be. Furthermore, as the duration of having diabetes increases, poor adherence to medication decreases; so medication adherence is stronger among those who have had diabetes longer. Figure 2b demonstrates that as the age group of patients with diabetes increases, both poor control of the disease and poor medication adherence decreases. In other words, control and adherence are stronger among the older age groups.

A closer look at the relationship between the primary predictors indicates that while the proportion with good adherence increases by each increasing disease duration group (40.5%, 43.4% and 48.2%, respectively), the proportion with poor control also increases (9.9%, 12.6% and 19.0%, respectively) with disease duration (Figure 2a). In contrast, while adherence increases with age, poor control decreases in the older age groups (Figure 2b).

Consequently, we further examined the association of adherence with disease duration and glucose control in iterative adjusted models (results not shown). There was no evidence of a mediating role of medication adherence between disease duration and glucose control; taking the variable out of the model changed the log odds by less than 10%. The interaction term of duration group*adherence level was statistically significant (p<0.0001) in the full model. However, only two of the nine comparisons within the duration group*adherence level interaction term were significant and when the model was stratified by duration groups, there were only minor variations in the strengths of association. The association between poor medication adherence and poor glucose control was similar across short (OR = 2.6; CI: 2.4–2.9), medium (OR = 2.9; CI: 2.7–3.2) and long (OR = 2.4; CI: 2.3–2.5) duration groups.

In the subsequent multivariate model, it was indicated that medication adherence levels did indeed mediate the association between age and glucose control; differences in the log odds were most markedly observed in the youngest age group (<30 years) and somewhat among those aged 30–54. Minimal differences were seen among those aged 55+. In other words, poor medication adherence seems to play a significant role among younger people in determining their high rates of poor glucose control, while playing a minor or no role in driving poor glucose control among older adults.

Furthermore, the population attributable risk (PAR) for poor glucose control due to poor adherence persisted across all age groups, and was particularly notable among younger individuals. Comparing the poorly adherent in the two youngest age groups (<30 and 30–54 years) to highly adherent (>80%), there would be a corresponding 54% and 37% attributable risk for poor glucose control among the respective groups. While this improvement in glucose control was found across all age groups, the PAR exhibited a decreasing linear trend with age (p<0.001), dropping to 14% in the 75+ age bracket. If the poorly adherent with shorter-term illness (<5 years) would become highly adherent (>80%), the corresponding PAR for poor glucose control would be 35%. However, among patients with longer-duration illness (another key risk group for low adherence) the corresponding PAR for poor glucose control would be 24%.

## Discussion

Although OAMs are considered efficacious in helping diabetes patients to control their glucose, reported levels of ‘good adherence’ to drug therapy vary greatly between 38–84% in several populations studied [9]–[15]. When considering physician prescribed regimens, we found relatively low overall adherence rates (less than 50% of the population found to be 80% or more adherent to their OAMs) among the 228,846 adult diabetes patients in our study. Inadequate medication adherence was found to have a 27% attributable risk for poor glycemic control. This figure, however, varies considerably from a 54% attributable fraction in patients younger than 30 years of age, to 14% in patient 50 years and older. Additionally, 24% of the risk for poor glycemic control is attributable to inadequate medication adherence in the subgroup of patients with longer duration illness.

Our study expands on the current evidence establishing the importance of medication adherence in contributing to poor glycemic control in different sub-groups of a large, general adult diabetes population, achieved through the utilization of an objective adherence measure that incorporates pharmacy dispensing and written prescriptions. We demonstrated that low medication adherence plays a clear mediating role in explaining high rates of poor glucose control, particularly among younger adult diabetes patients, thereby, identifying a key target population for intervention.

Many of the past studies evaluating this relationship for poor glucose control have failed to support findings with statistically significant results, mainly due to small sample sizes, as well as to the subjectivity in the adherence measures used [23]–[24]. Two large-scale studies have reported an inverse association between medication adherence and poor glycemic control in large populations; however, one study was comprised predominantly of male veterans and, thus, was not representative of a general population [16], and the other did not establish the relationship in an adjusted model [6]. Our results overcome these methodological caveats and confirm that there is a strong inverse association between medication adherence and poor control, with patients who are poorly adherent to their OAMs (<50%) being nearly three times more likely to have poor glycemic control than patients who are adherent (>80%).

Prominent subgroups most vulnerable for poor glucose control in our study were younger patients (<55 years) and those with longer duration of diabetes (>5 years). A study that examined the causes of poor glucose control and tested for the mixed effects of age obtained similar findings, but failed to show a statistically significant association between age and poor glucose control [31]. Two other studies [16], [22] also showed a positive association between younger age and poor glucose control, but neither assessed whether poor medication adherence had a role in explaining this association. We evaluated the role of medication adherence in our model and found that it was a strong mediator of poor glycemic control among younger individuals with diabetes. A recent study in the United States reported poorer rates of medication adherence among lower age groups (age 18–64); however, these were not evaluated in a multivariate model and were based on self-reported adherence levels [13]. Furthermore, when calculating the attributable fraction, we observed that a greater proportion of poorly controlled younger diabetes patients, compared to older age groups, could be prevented if they would be at least 80% adherent to their OAMs.

Longer disease duration has also been reported in a number of studies as an independent predictor of poor glycemic control among diabetes patients; yet, again, none of these studies investigated the role of medication adherence in this association [5], [11], [19], [25], [31]. Our results confirm that patients with disease duration of more than five years are at greater risk of poor glycemic control compared to those with shorter duration of illness. Higher risk of poor control exists even though the longer disease duration patients are more likely to have improved medication adherence, an independent factor inversely associated with poor control. This suggests that the protective effect of improved medication adherence among these longer-standing diabetes patients is negated by other factors that contribute to poor glycemic control. The phenomenon of poor glucose control in patients with disease of long duration has been attributed to islet cell exhaustion, i.e. decreasing pancreatic function, and subsequent lower levels of secreted insulin [32]. This, in combination with insulin resistance, which is characteristic in type 2 diabetes, yields the worsening of glucose control over time [32].

As many diabetes patients are taking multiple chronic medications, which are actively managed by physicians, some of these medications will only be prescribed for intermittent periods. Consequently, when examining adherence to multiple medications, the ability to relate prescriptions filled to prescriptions written, as done in this study, may likely be critically important. Additionally, the variation among studies in measurements of medication adherence may substantially influence the strength of its association with glucose control. Many studies have used subjective patient-reported assessments of adherence [11], [13], [19], [26] rather than quantitative measures, such as medication possession ratios (MPR). Although self-reported medication adherence is a widely accepted measure in research, there are well-documented biases and errors in member-reported adherence measures [24], [33]. The novel measure we utilized to evaluate weighted adherence to multiple medications was capped by first and last prescriptions dispensed, and detects those patients who were written a prescription but did not fill it. This potentially provides a more accurate indication of the complete medication period intended by the physician [30]. Previous studies which have objectively measured adherence for multiple medications, such as Choudry et al.'s [34], do not utilize written prescription data (only filled prescriptions), and therefore may overestimate poor adherence because of the inability to capture physician intended treatment gaps. Therefore, the MWA measure in our study potentially captures these varying treatment regimens and the active medication management of physicians more accurately than traditional MPR measures.

There are several limitations to our study. Given the data limitations for examining long duration of illness (>5 years) mentioned above, we lack a more nuanced understanding of the association between those with, for example, over 10–15 years of disease exposure and glycemic control. We were also unable to consider smoking status, given incomplete data on this variable among those over age 75. Consequently, we did not include the variable in the analyses which could potentially yield an omitted variable bias, thereby over or under-estimating the effects of the other factors in the model. Additionally, while the study was intended to focus on type 2 diabetes, our database does not distinguish between type 1 and type 2 diabetes; however, by restricting the study sample to adult diabetes patients taking OAMs, we eliminated the majority of the individuals with type 1 diabetes from the sample. Lastly, a limitation of our weighted measure of adherence relates to the dosage/time details of the prescription. In CHS, the standard prescriptions for chronic medications are issued for a 30-day supply. The MWA is based on this standard; therefore, to the extent that prescriptions are written to cover more than a 30-day supply, there will be discrepancies between written and filled prescriptions over periods of time that are tabulated as reduced adherence.

In summary, we have tested, in a large-scale population-based study, the association between poor adherence to diabetes medications and poor glucose control among several subgroups of adults with diabetes. Poor medication adherence was a key mechanism in explaining why younger adults with diabetes have poor glycemic control, with adherence making up a highest attributable fraction of poor control in this sub-segment. This suggests that interventions for addressing medication adherence may prove to be particularly beneficial in helping younger diabetes patients achieve greater glucose control.
